# Construction of rice supply chain supervision model driven by blockchain smart contract

**DOI:** 10.1038/s41598-022-25559-7

**Published:** 2022-12-05

**Authors:** Xiangzhen Peng, Xin Zhang, Xiaoyi Wang, Haisheng Li, Jiping Xu, Zhiyao Zhao

**Affiliations:** 1 Beijing Key Laboratory of Big Data Technology for Food Safety, Beijing Technology and Business University, Beijing, 100048 China; 2 Key Laboratory of Industrial Internet and Big Data (Beijing Technology and Business University), China National Light Industry, Beijing, 100048 China; 3 Beijing Institute of Fashion Technology, Beijing, 100048 China

**Keywords:** Computer science, Information technology

## Abstract

The outbreak of the COVID-19 and the Russia Ukraine war has had a great impact on the rice supply chain. Compared with other grain supply chains, rice supply chain has more complex structure and data. Using digital means to realize the dynamic supervision of rice supply chain is helpful to ensure the quality and safety of rice. This study aimed to build a dynamic supervision model suited to the circulation characteristics of the rice supply chain and implement contractualization, analysis, and verification. First, based on an analysis of key information in the supervision of the rice supply chain, we built a dynamic supervision model framework based on blockchain and smart contracts. Second, under the logical framework of a regulatory model, we custom designed three types of smart contracts: initialization smart contract, model-verification smart contract, and credit-evaluation smart contract. To implement the model, we combined an asymmetric encryption algorithm, virtual regret minimization algorithm, and multisource heterogeneous fusion algorithm. We then analyzed the feasibility of the algorithm and the model operation process. Finally, based on the dynamic supervision model and smart contract, a prototype system is designed for example verification. The results showed that the dynamic supervision model and prototype system could achieve the real-time management of the rice supply chain in terms of business information, hazard information, and personnel information. It could also achieve dynamic and credible supervision of the rice supply chain’s entire life cycle at the information level. This new research is to apply information technology to the digital management of grain supply chain. It can strengthen the digital supervision of the agricultural product industry.

## Introduction

Rice is one of the most important food crops in the world^1,2^. Rice is grown in 122 countries around the world and is closely related to human development^3^. Most research on rice has focused on rice planting. For example, hybrid rice is used to increase rice yield, and transgenic technology is used to increase the cold resistance and heat tolerance of rice. In recent years, health and safety problems related to the quality and safety of rice have frequently arisen around the world^4,5^. Examples include heavy metal pollution, pesticide pollution, mycotoxin pollution, and excessive additives. The above situation indicates that the rice supply chain urgently needs to use information technology to achieve high-quality supervision of rice quality and safety and improve rice quality and safety.

Traditional supervision methods for improving the rice supply chain mainly involve strengthening the legal awareness of rice-production personnel, enhancing the reporting of rice-safety incidents, establishing technical standards for rice-production safety, and increasing government supervision^6–8^. Although traditional supervision methods have played a positive role in the supervision of the rice supply chain, they are labor and material intensive. In addition, traditional supervision also has the characteristics of low data security and complex processes required by supervision. Most research on the supervision of rice supply chains based on blockchain has focused on the supervision of a single link, and there is a lack of supervision covering the entire life cycle of the supply chain^9^. Moreover, the rice-circulation process is dynamic, and most existing research has involved static supervision of the rice supply chain. Therefore, while existing blockchain-based supervision methods break the traditional grains and oils supervision model to a certain extent, they lack intelligent dynamic supervision.

Blockchain is a distributed ledger technology that broadcasts in the form of P2P. Therefore, blockchain is characterized by decentralization, unchangeable content and traceable information. Smart contract is a typical representative of blockchain 2.0, which is essentially a piece of code that can run automatically and self check. The smart contract extends the application scenario of the blockchain, and the blockchain ensures the code security of the smart contract. Finally, blockchain smart contract technology was formed^10–12^.

In this study, we designed a dynamic supervision model for the rice supply chain based on blockchain and smart contracts that could achieve the dynamic supervision of the rice supply chain. The highly automated and intelligent design of these smart contracts makes the entire supply chain more secure and intelligent. The prototype system we established can provide companies in the rice supply chain with internal management and data interconnection functions. It can give regulators a fast, effective way to dynamically supervise the entire supply chain, dynamically supervise the regulators themselves, and provide consumers with services such as rice traceability.

### Contribution

We first conducted an overall analysis of the rice supply chain and established a dynamic supervision model based on blockchain and smart contracts. By customizing smart contracts, we enabled the model to automatically collect information and supervise the rice supply chain. On that basis, a prototype system for the dynamic supervision of the rice supply chain was established. Generally, this research can be divided into three parts:

- We used blockchain as the technology platform to provide a credible environment for the supervision of the rice supply chain. The scope of supervision covers the entire life cycle of rice. This can achieve the interconnection and intercommunication of data between all links of the rice supply chain and various departments.
- By customizing and compiling dynamic smart contracts, we achieved the automatic collection of data for the entire rice supply chain, the dynamic real-time supervision of supply-chain data, and the supervision of supervisors.
- After repeated project iterations, the proposed model can intelligently extract and integrate the collected data, providing a credible data source for subsequent supervision.

### Organization

The rest of this paper is organized as follows. The next part discusses the theoretical background. “Regulatory model construction” briefly introduces the model, while “Smart-contract design” presents the pseudo-code implementation of the contract. “Results and analysis” presents the results, analyzes the suitability of the prototype system using an example, and explains the practicality of the model. “Conclusion and future work” concludes.

## Related works

Strengthening the supervision of the quality and safety of rice is one of the primary ways to ensure food safety^13^. Traditional supervision models have been optimized to improve the level of rice supervision, mainly from the perspectives of ethics, law, technology, and propaganda. In terms of ethics, the moral awareness of production and processing personnel has been improved to reduce the occurrence of problems in the rice supply chain. The legal aspect has involved improving rice supervision laws and regulations, strengthening supervision, and conducting legal education. In terms of technology, it has been noted that the monitoring of heavy metals should be strengthened by establishing a biogeochemical cycle model, studying the sources of heavy metal pollution in rice fields, and identifying the migration of heavy metals between different rice paddies. Regarding publicity, rice supervision can be strengthened by publicizing the harmfulness of compromised rice and incorrect production and processing practices. Although such measures have strengthened the supervision of the rice supply chain to a certain extent, the supply chain has a huge amount of data and information, and traditional supervision systems mostly store data in a centralized manner, which is vulnerable to data tampering and loss.

Blockchain technology originated from Bitcoin and was mainly used in the financial field. Then, smart contracts were applied to blockchains, allowing for the encapsulation of business logic. Since then, blockchains have been extended into the domains of law, medicine, and food, among others. In the food domain, blockchain technology is used for food traceability and supervision because of its decentralized architecture and fully trusted operating environment (Table 1). Blockchain can ensure that nodes jointly maintain data security and that the entire data chain is traceable. In terms of data security, blockchain technology uses asymmetric encryption algorithms to encrypt data to ensure the security of data transmission.

**Table 1:** Application of blockchain technology in the field of food safety.

Category	Work content	References
Theoretical research	Theoretically apply blockchain to the food domain to verify its feasibility and practicality	^14–18^ ^19–22^
Research on integration with technologies such as the Internet of Things and smart contracts	Explore the application of blockchain, Internet of Things, smart contracts, and other new technologies to better solve problems in the food domain	^23–27^ ^28,29^
Food traceability research	Using the traceability and nontampering characteristics of blockchain, study the blockchain-based application of food traceability, establish models, and develop related systems to achieve blockchain implementation	^30–34^ ^35–38^
Food regulatory research	Using blockchain’s decentralized architecture, nontamperable nature, anonymity, and other characteristics, study blockchain-based food supervision and change the traditional food supervision model from a technical standpoint. Establish models and develop systems to achieve blockchain implementation	^39–42^

In terms of theoretical research, previous studies have mainly applied the principle of blockchain to the food field through the method of literature review. The feasibility and practicability after application are verified from the theoretical point of view. Through systematic review of literature and content analysis, researchers pointed out that blockchain can help improve food traceability, information transparency and recall efficiency^14,15,22^. As a new generation of distributed storage architecture, blockchain can improve system transparency^16^, accountability^17^ and auditability^20,21^ in the food field compared to traditional centralized systems. For example, Katsikouli et al. pointed out through research that the use of blockchain-based systems to manage supply chains provides significant benefits, such as faster and more reliable traceability^18^. Li et al. analyzed the development and changes of blockchain technology in the agricultural industry chain from the source to the operation, production, service, safety, security and other aspects. They pointed out that blockchain can bring good supervision and traceability to agricultural e-commerce^19^.

In terms of integrated applications with technologies such as the Internet of Things and smart contracts. In previous studies, scholars have conducted research on the integration and application of blockchain and machine learning, Internet of Things^27–29^, artificial intelligence^26^ and other technologies. It can be used to solve the problems faced by the blockchain itself, as well as improve the role of the blockchain in the food field. For example, Wang et al. used oversampling techniques to populate sample feature data of imbalanced Ponzi-like smart contracts. LSTM models are trained by learning from feature data for future Ponzi scheme detection^23^. Capocasale et al. proposed a solution to integrate blockchain and IoT by exploiting the enhanced data security and integrity provided by NB-IoT^24^.

In terms of food traceability research, in previous studies, scholars have used the traceability and non-tampering characteristics of blockchain^32^, study the blockchain-based application of food traceability, establish models, and develop related systems to achieve blockchain implementation^34,35^. For example, Tian et al. constructed an agri-food supply chain traceability system for trusted traceability of agricultural product information^31^. Mondal et al. pointed out that the blockchain architecture facilitates the creation of a tamper-proof digital database of food packaging in each instance^38^. In previous studies, scholars have used blockchain to assist in information traceability in scenarios such as dairy products^30^, agricultural farming^33^, cold chain traceability^37^, and rice^36^.

In terms of food supervision research, in previous studies, scholars have used blockchain’s decentralized architecture, nontamperable nature, anonymity, and other characteristics, study blockchain-based food supervision and change the traditional food supervision model from a technical standpoint. Establish models and develop systems to achieve blockchain implementation^39,40^. For example, Hao et al. proposed a food market supervision method based on blockchain and deep learning models^41^. Tao et al. proposed a hierarchical multi-domain blockchain (HMDBC) network structure and a secondary inspection mechanism to solve the problems of incomplete on-site supervision and delayed disposal in the traditional food supervision system^42^.

Applying blockchain to the supervision of the rice supply chain is a technical innovation that can improve the current status of supply-chain supervision. It ensures the security of data in the rice supply chain and achieves the credible traceability of rice^43–45^. Existing research in this area has the following shortcomings, which this study aimed to overcome:

- The research has small coverage, limited to a single link of supervision, and lacks supervision of the whole life cycle.
- The degree of supervision intelligence is low. Although manual operation is reduced to a certain extent, a large amount of manual operation is still required for data sorting and collection.
- The circulation of the rice supply chain dynamically changes, and existing supervision technologies cannot achieve dynamic supply-chain supervision.
- The ability to supervise and manage supervisor behavior is insufficient.

## Regulatory model construction

### Analysis of supervision information in the rice supply chain

The supply chain research of different crops in the agricultural and food industry has been widely concerned by scholars. In previous studies, Salehi et al. designed a new closed-loop supply chain for the walnut industry, including the forward flow of farms, procurement centers, separation centers, etc. Collection and recycling centers and secondary markets flow in opposite directions^46^. Chouhan et al. Provides a closed-loop supply chain for the sugarcane industry, including producers, processing units (or sugarcane industry), distribution units, retailers, other industries, fertilizer units, and fertilizer markets^47^. Salehi et al. designed a closed-loop supply chain network for the avocado industry^48,48,50^. We refer to previous scholars' research on agricultural product supply chain to analyze the rice supply chain.

Given current shortcomings in the supervision of rice supply chains, we designed a dynamic supervision model based on blockchain and smart contracts. This model serves all participating companies, consumers, and supervisors in the rice supply chain. There are two types of monitoring data in this dynamic supervision model. The first type contains information about the following: heavy metals, mycotoxins, pesticide residues, pests, fumigants, herbicides, and other toxic, harmful pollutants in the supply chain; seed sources, product batches, packaging materials, storage time, and transportation information; and seed-price transaction records, fertilizer prices, purchase prices, labor costs, and the dressing and hygiene requirements of all of the participants in the link (Table 2). The second type of data is related to dynamically monitoring the behavior of participating enterprises, consumers, and regulators (Table 3). This model can provide a dynamic supervision method and reliable data source for the rice supply chain.

**Table 2:** Classification table of key information of the whole rice supply chain.

Rice supply chain		Basic information	Hazard information	Transaction record
Plant		Seed source, production site, planting/harvesting time, rice yield rate, fertilizer/pesticide use information	Mycotoxins, heavy metals, pesticide residues, pests, and diseases	Seed price, fertilizer price, labor cost, total cost, sales price
Purchase and storage	Acquisition	Purchase batch, purchase inspection report	Mycotoxins, fumigants, and herbicide residues	Purchase price, labor cost
	Dry	Product batch, drying record report		Drying (e.g., equipment)/labor cost
	Edulcoration	Product batch, pharmaceutical use record, impurity content, impurity removal rate		Miscellaneous removal (e.g., pharmaceuticals, equipment)/labor costs
	Warehousing	Inventory number, product batch, product source, storage time, outgoing time, quality inspection report, product category, product quantity		Warehousing (e.g., warehouse, tools)/labor cost, warehousing price
Processing	Ridge valley	Product batch, ridged grain method, equipment inspection record, ridged grain time, roughness removal/hulling rate	Mycotoxins, heavy metals	Ridge valley (e.g., equipment)/labor cost
	Rice milling	Product batch, rice milling method, equipment inspection record, whole rice/broken rice rate		Rice milling (e.g., equipment)/labor cost
	Color selection	Product batch, color sorting accuracy, take-out ratio		Color sorting (e.g., equipment)/labor cost
	Polishing	Product batch, polishing method, polishing rate		Polishing (equipment)/labor cost
	Packing	Product batch, packaging material source, packaging material qualification certificate, product quality information		Packaging cost/labor cost, price of first finished product
Transport		Product batch, transportation vehicle information, vehicle disinfection report, departure place, route, arrival time, driver information	Fungi and toxins produced by mildew caused by abnormal temperature and humidity	Transportation cost
Storage		Product batch, temperature and humidity record, product source, storage time	Mycotoxins	Warehousing cost, outbound price
Sale		Product name/batch, product integrity rate, purchase time, sale time, sales address, product quantity	None	Purchase price, sales price

**Table 3:** Permission data collection table.

Collection object	Permission data information
Participating companies	Company name
	Company address
	Corporate contact
	Business license
	Main business
	Legal representative
	Registered capital
	Enterprise nature
	Credit data
Regulatory authority	Institution name
	Department
	Supervision link
	Link standard description
	Rules and regulations
	Prevention and control strategy
	Credit data
Consumer	Identity information
	Contact information
	Purchase history
	Participation record
	Credit data

### Model

**Figure 1:**
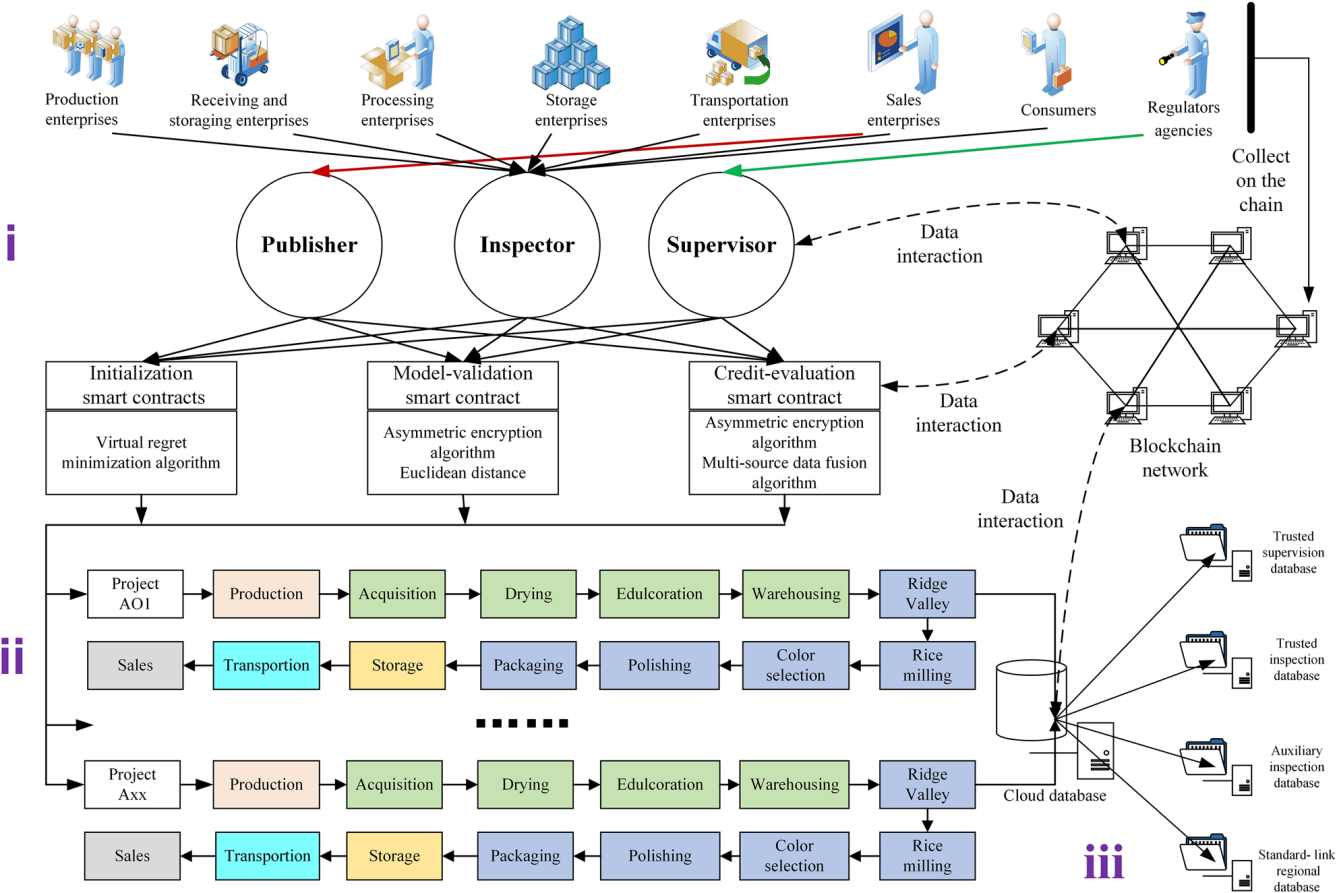
Dynamic supervision model for the rice supply chain.

Figure 1 shows the dynamic supervision model for the rice supply chain. It is divided into an initialization module, a supervision module, and a storage module. The initialization module divides supply-chain participants into three categories: enterprises, consumers, and regulatory agencies. Enterprises are divided into six categories: production, receiving and storing, processing, storage, transportation, and sales. The receiving and storing link includes four subnodes: acquisition, drying, edulcoration, and warehousing. The processing node has five subnodes: ridge valley, rice milling, color selection, polishing, and packaging. The supervision module dynamically supervises rice data through customized smart contracts. The storage module mainly displays the storage method for data, which are stored and recalled through the storage mode of “blockchain + cloud database.” The data in this paper comes from the collection of IoT devices such as RFID, NFC, mobile phones, computers, and GPS. The model-verification smart contract is designed to store data on the blockchain. The rice supply chain dynamic supervision model established by blockchain and smart contract technology is used to analyze and process the collected data, thereby realizing the dynamic supervision of the data.

- Initialization module

The model is first tested, and all of the participants are divided into three categories after system certification: publisher, inspector, and supervisor. The publisher is a sales company and can broadcast rice demand information to other link nodes in the chain. Inspectors are all participants, except the supervisory agency, who can assist the supervisory institution in the real-time dynamic inspection of the supply chain and support the model in the real-time review of the supervisory agency’s behavior. The supervisor is the supervisory institution participating in the rice business, which can supervise supply-chain information as well as the behavior of inspectors. Mutual supervision between the supervisor and the inspector ensures the credibility of supervision, forming a two-way supervision mechanism for rice safety and quality.

(b) Supervision module

The supervision function of the model depends on mutual supervision between smart contracts, which is mainly reflected in the logical design of the contracts. There are three smart contracts in the model: the initialization, model-verification, and credit-evaluation smart contracts. This achieves the mining and extraction of data in each link of the supply chain and helps inspectors and supervisors distinguish trustworthy and untrustworthy personnel. The contribution coefficient guides the formation of the trusted supervision database, trusted inspection database, auxiliary inspection database, and standard-link regional database. Blockchain is the bottom layer of the supervision model, providing a decentralized environment for the model and ensuring its credibility. The model stores the data index in the blockchain network to achieve data interaction. Data in the blockchain network are extracted through smart contracts, and the data are then queried and supervised. The main model information is stored as cloud data, and the cloud database is divided into four subdatabases, which store different data types.

(c) Storage module

The rice supply chain supervision model forms four supervision databases based on blockchain and smart contracts: trusted supervision database, trusted inspection database, auxiliary inspection database, and standard-link regional database. The trusted supervision database is composed of credible supervision models selected from those of the rice supply chain. The trusted inspection database is an auxiliary supervision database that inspects the entire supply chain in the business process. It is a collection of credible inspection models formed by smart contracts based on the contribution coefficient of the inspectors. The auxiliary inspection database is a database for consumers. It is a collection of models generated after consumers have completed the inspection work, playing a certain role in the supervision of the supply chain. The standard-link regional database is a collection of models whose content, selected from all of the links in the supply chain, meets national standards. After the model runs a business once, a set of trusted data is generated and stored in four different types of subdatabases. After repeated business operations, the data stored in the database is updated and optimized accordingly. After a certain number of operations, the database can provide a credible data source for the rice supply chain supervision model and achieve its multifaceted supervision.

## Smart-contract design

**Figure 2:**
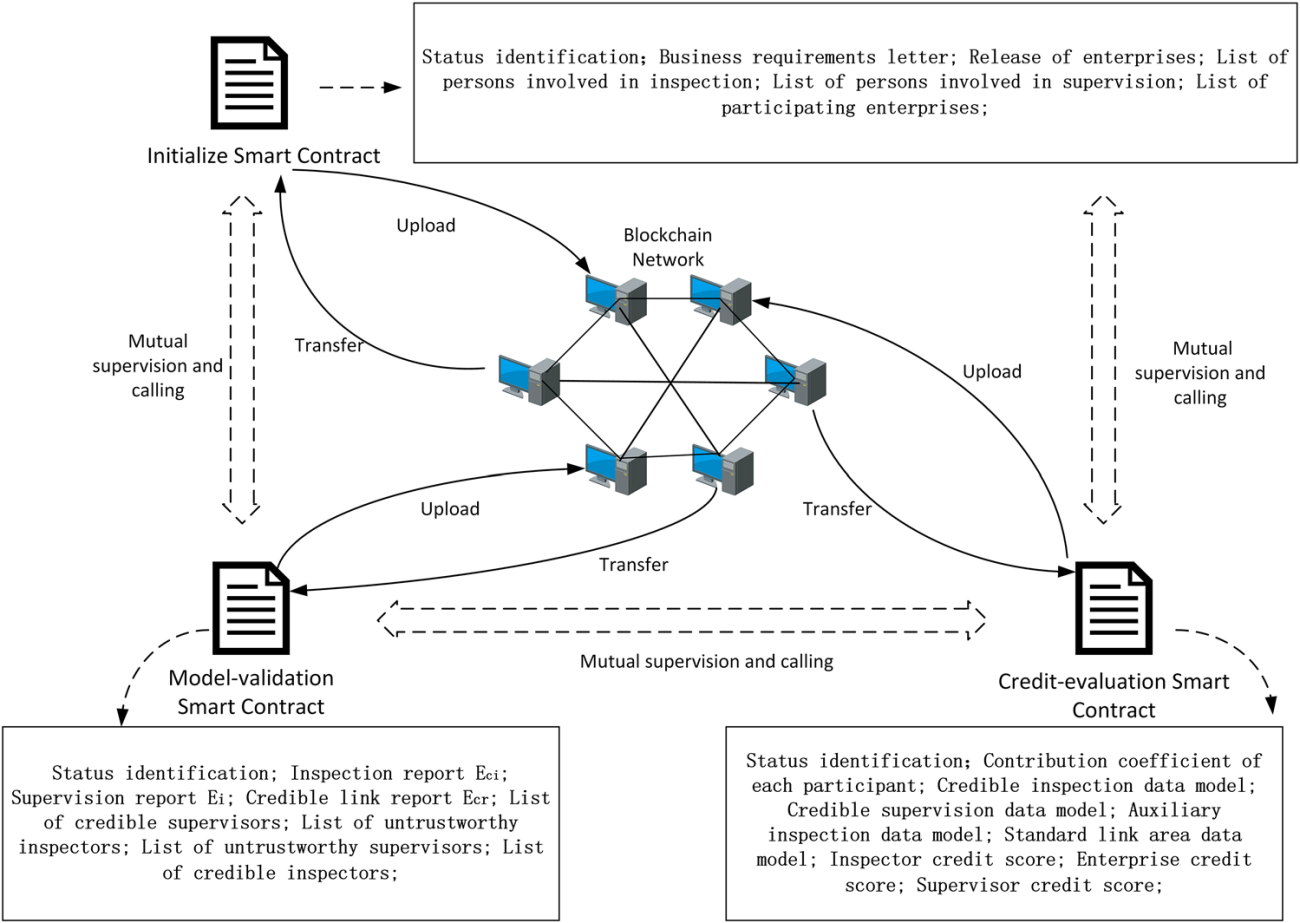
Contract design diagram.

We edited initialization, model-verification, and credit-evaluation smart contracts to suit the purposes of the proposed dynamic supervision model. Using the relationship of mutual supervision and calling between contracts ensures that the model can automatically collect and analyze data, enabling it to dynamically monitor the life cycle of the rice supply chain in real time. The contract design module is the core module whereby the model can be realized. As a distributed ledger based on a P2P network, blockchain provides a trusted environment for the automatic execution of smart contracts. Figure 2 shows the smart-contract design.

The smart-contract design serves to realize model functions. The smart contract in this study guarantees the authenticity of the data source by uploading and calling data in the blockchain. It uploads data collected by the model and the interaction information between contracts to the blockchain network to ensure data security. The contracts call and supervise each other.

Smart contracts are the core part of the dynamic supervision model for the rice supply chain. This study designed pseudo-code for smart-contract initialization, model verification, and contribution evaluation. The operation logic of the model is encapsulated by setting relevant reservation rules to achieve the overall function of the model. The smart contract that the model runs once has a sequence of rules. This study used two unsigned flags to match the sequence of calls—{start I, end O} = {link, link}, {start I, end O} = {link, break}, and {start I, end O} = {break, break}—where link is true and break is false, corresponding to the initialization, model-verification, and credit-evaluation smart contracts.

The definition and function description of each contract are as follows:

### Initialize smart contract

Initializing the smart contract is the initial stage of the model’s supervision of the rice supply chain. The contract first initializes the model, which mainly authenticates the business release personnel and the release of the business. It automatically screens participating companies that meet the preset conditions and determines the inspectors and supervisors participating in the batch. Finally, the task distribution of inspection and supervision is achieved. The virtual regret minimization algorithm is embedded in the contract, which can solve the hedging problem of the participants' business. It guarantees a fair distribution of rice business.

First, all of the participating companies, consumers, and regulatory agencies register on the chain, and the sales companies publish mission requirements and broadcast them to other nodes through the P2P network. The task requirement letter contains two parts. One part is rice demand information, and the other part is the minimum credit points required by companies, consumers, and regulatory agencies participating in this batch of the sales company. Equation (1) shows the calculation of the credit score of the enterprise. Each participant requests participation after viewing the requirements and then initializes the smart contract to automatically verify, approve, and output. In the initial stage, a rice project and three participating lists are output: the list of participating companies, list of participating inspectors, and list of participating supervisors, denoted as *F*_*list*_, *C*_*list*_, and *R*_*list*_, respectively.

1 $$H_{F} = H_{1} + H_{2} + H_{3} + H_{4} + H_{5} + \cdots + H_{{\text{n}}} ,$$

where *H*_*i*_ is the individual credit score of the personnel participating in the inspection work in the enterprise, *F* is the participating company, and the credit score of *F* is reflected in the cumulative sum of the credit scores of personnel participating in the inspection work in the enterprise.

After the sales company is identified as the business issuer and publishes the task requirements to the blockchain network, each participant asks to participate in the task. The smart contract is initialized for credit score screening, and the participating personnel or companies after the screening are approved as eligible to participate in the business. Participants can apply for participation requests for multiple projects at the same time. Since the credit scores of the participants are updated after each business, a participant can only participate in the supervision or inspection of the business one at a time. When the person passes the credit score review of multiple businesses at the same time, a virtual regret minimization algorithm (CFR) is used to solve the attribution problem of the participant.

CFR belongs to an incomplete information game; that is, without traversing the regret value of all of the nodes, the virtual regret value is calculated through the simulation calculation process, and the strategy with the smallest regret value is selected as the optimal strategy for the game. In this way, a Nash equilibrium state is reached, and one party wins ownership of the participant.

This study used the CFR algorithm to provide the optimal response strategy for each publisher. In the strategy combination, the strategy of each publisher is the optimal reaction strategy relative to other publishers; that is, the strategy combination is the Nash equilibrium strategy. With the CFR algorithm, there is no need to traverse the regret value of all of the nodes; thus, each publisher can quickly obtain the optimal response strategy under the condition of limited computing resources. Each publisher adopts their own optimal response strategy to play the game to determine the attribution of the participant. The specific design steps are as follows:

- Rule description

Set A is defined as the set of actions that all publishers who need to participate in the game can take, including two states of “raise” and “abandon,” represented by *a* and *b*, respectively. Set B is the set of information defined as the rules of this game and the historical behaviors taken by the publisher. *A (B)* represents the set of behaviors that can be taken under information set *B*. Behavior *a*_*i*_ $\in$ *A (B*_*i*_*)* taken by publisher *I* in the t round reflects strategy ${\sigma }_{i}^{t}$ taken by the publisher in the *t* round. The strategies adopted by all publishers in the t round of behavior a constitute a set of strategy combinations ${\sigma }^{t}$. The strategy reflected by taking action a under set *B* is denoted as ${\sigma }_{B\to a}$. The actions taken by all of the publishers in round *t* is a sequence denoted as *h*. Take a certain strategy σ to calculate the occurrence probability of action sequence *h* and record it as $\pi^{\sigma } (h)$*.* The probability of all action sequences that can reach information set *B* is accumulated as the probability of occurrence of information set *B*, as shown in Eq. (2):

2 $$\pi^{\sigma } (B) = \sum\nolimits_{{{\text{h}} \in B}} {\pi^{\sigma } (h)} ,$$

where $\pi^{\sigma } (B)$ is the probability of the occurrence of information set *B*. Given the ending situation *z* $\in$ *Z* of the game, the publisher’s income after the end of the game is recorded as *u*_*i*_(*z*). The probability of reaching the final position *z* after applying the game action sequence *h* under the strategy combination $\sigma$ is $\pi^{\sigma } (h,z)$.

(b) Calculation of virtual regret

When the publisher adopts strategy $\sigma$, the virtual value of the corresponding action sequence *h* is expressed as $v_{i} (\sigma ,h)$, as shown in Eq. (3):

3 $$v_{i} (\sigma ,h) = \sum\limits_{z \in Z} {\pi_{ - i}^{\sigma } } (h)\pi^{\sigma } (h,z)u_{i} (z).$$

The virtual regret value obtained by publisher *i* by taking action *a* based on action sequence *h* is expressed as $r(h,a)$; the calculation is shown in Eq. (4):

4 $$r(h,a) = v_{i} (\sigma_{B \to a} ,h) - v_{i} (\sigma ,h).$$

The calculation of the regret value of information set *B* corresponding to action sequence *h* is shown in Eq. (5):

5 $$r(B,a) = \sum {r(h,a)} .$$

The regret value of issuer *i* taking action *a* in the *T* round is shown in Eq. (6):

6 $${\text{Re}} gret_{t}^{T} (B,a) = \sum\limits_{t = 1}^{T} {r_{i}^{t} } (B,a).$$

Similarly, the situation where the regret value is negative is not considered, as shown in Eq. (7):

7 $${\text{Re}} gret_{t}^{T, + } (B,a) = \max (R_{i}^{T} (B,a),0).$$

In the *T* + *1* round, the probability that publisher *i* chooses action *a* is calculated as shown in Eq. (8):

8 $$\sigma_{i}^{T + 1} (B,a) = \left\{ {_{{\frac{1}{{\left| {A(B)} \right|}}\begin{array}{*{20}c} {} & {} & {} & {} & {\begin{array}{*{20}c} {} & {} \\ \end{array} \begin{array}{*{20}c} {} & {\begin{array}{*{20}c} {} & {} \\ \end{array} \begin{array}{*{20}c} {} & {otherwise} \\ \end{array} } \\ \end{array} } \\ \end{array} .}}^{{\frac{{{\text{Re}} gret_{t}^{T, + } (B,a)}}{{\sum {a \in A(B){\text{Re}} gret_{t}^{T, + } (B,a)} }}\begin{array}{*{20}c} {} & {} \\ \end{array} if\sum\nolimits_{a \in A(B)} {{\text{Re}} gret_{t}^{T, + } (B,a)} > 0}} } \right.$$

After many simulations, the publisher finally determines the optimal decision and forms the corresponding optimal strategy group, which is to maximize the Nash equilibrium state to ensure that the game is completely fair and just.

**Figure 3:**
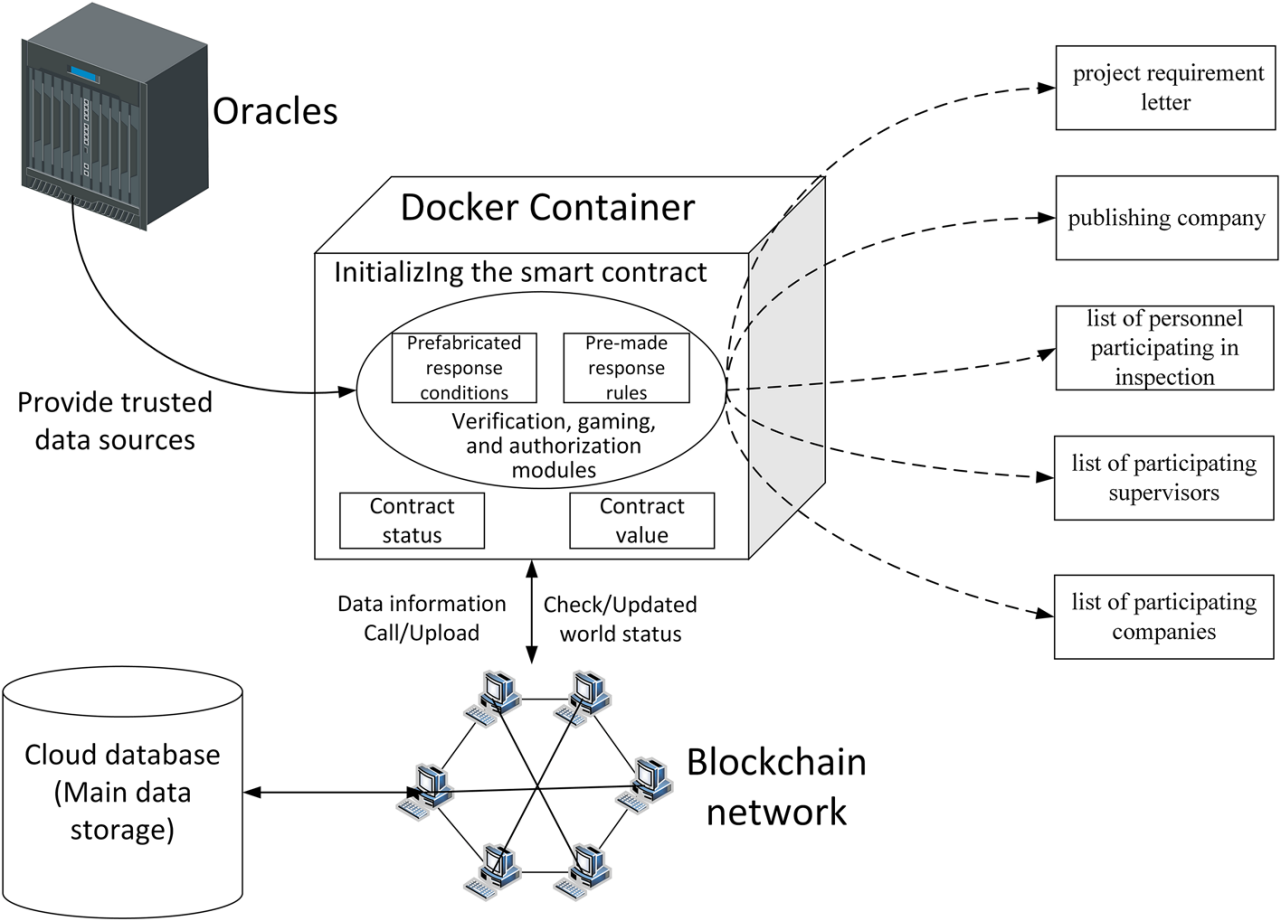
Logic diagram for initializing the smart contract.

The pseudo-code design for initializing the smart contract is shown in Algorithm 1. The detailed logic design of Algorithm 1 is in Appendix A. Figure 3 shows the deployment logic diagram of the initial smart contract.
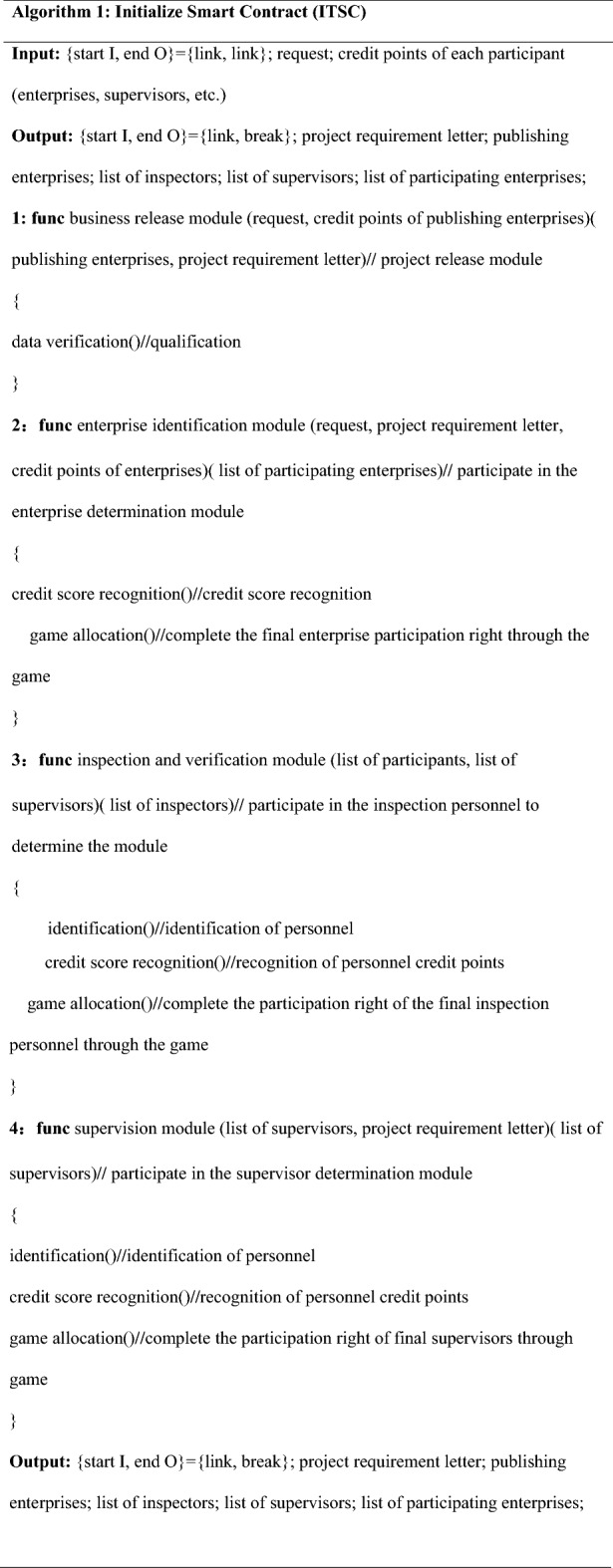

### Model-validation smart contract

The model-verification contract encapsulates the core function of the dynamic supervision of the rice supply chain and is the guarantee for achieving dynamic supervision. The contract first uploads business data in real time, distributes datasets to inspectors and supervisors, and submits assessment reports for them. Finally, the reports are quantitatively compared and analyzed through Euclidean distance, and credible/untrustworthy supervisors and inspectors are screened out.

After permissions are verified and distributed by the initialization smart contract, inspectors and supervisors assume inspection and supervisory powers. The logic of the model-verification link is as follows: first, the contract distributes the dataset—namely, the rice supply chain data corresponding to the contract distribution inspection\supervision link. The path of the contract distribution dataset is shown in Eq. (9):

9 $$Path_{file} = \{ RDS\{ D_{i} \{ H_{i} \{ file\} \} \} ,pk_{p} \} ,$$

where *RDS* is the cloud database, *D*_*i*_ is the library location of the project in the cloud database, Hi is the library where the inspector and the specific link needed for supervision are located, and *pk*_*p*_ is the public key of the person applying for the data. The model-verification smart contract uses the public key of the person applying for the data to encrypt and transmit the data, and the applicant uses the private key to decrypt and obtain the encrypted data.

Second, after inspectors and supervisors obtain the relevant datasets, inspectors *i* form an inspection report *E*_*ci*_ locally, and supervisors *i* form a supervisory report *E*_*i*_ locally. After that, the contract encrypts the report and transmits it to the blockchain network. The encrypted transmission method is shown in Eqs. (10) and (11):

10 $$Send_{{E_{ci} }} = \{ W_{i} ,Y_{i} ,Z_{i} \}_{{sk_{p} }} ,$$

11 $$Send_{{E_{i} }} = \{ W_{i} ,Y_{i} ,Z_{i} \}_{{sk_{p} }} ,$$

where $W_{i} ,Y_{i} ,Z_{i}$ are the hazardous substance record\circulation record\related responsibility information of the *i*th link, respectively.

Then, the supervisor obtains inspection report *E*_*ci*_; the obtaining method is shown in Eq. (12). After credibility is determined, the certification signature is sent to the inspector. After the inspector obtains the certification of sufficient supervisors, the inspector first publishes trusted link report *E*_*cr*_ and then broadcasts it to other nodes.

12 $$Path = \{ RDS\{ D_{i} \{ H_{i} \{ E_{ci} \} \} \} ,pk_{p} \} ,$$

where the inspector uses the public key of the supervisor to encrypt the data, and the supervisor uses his or her own private key to decrypt the data.

**Figure 4:**
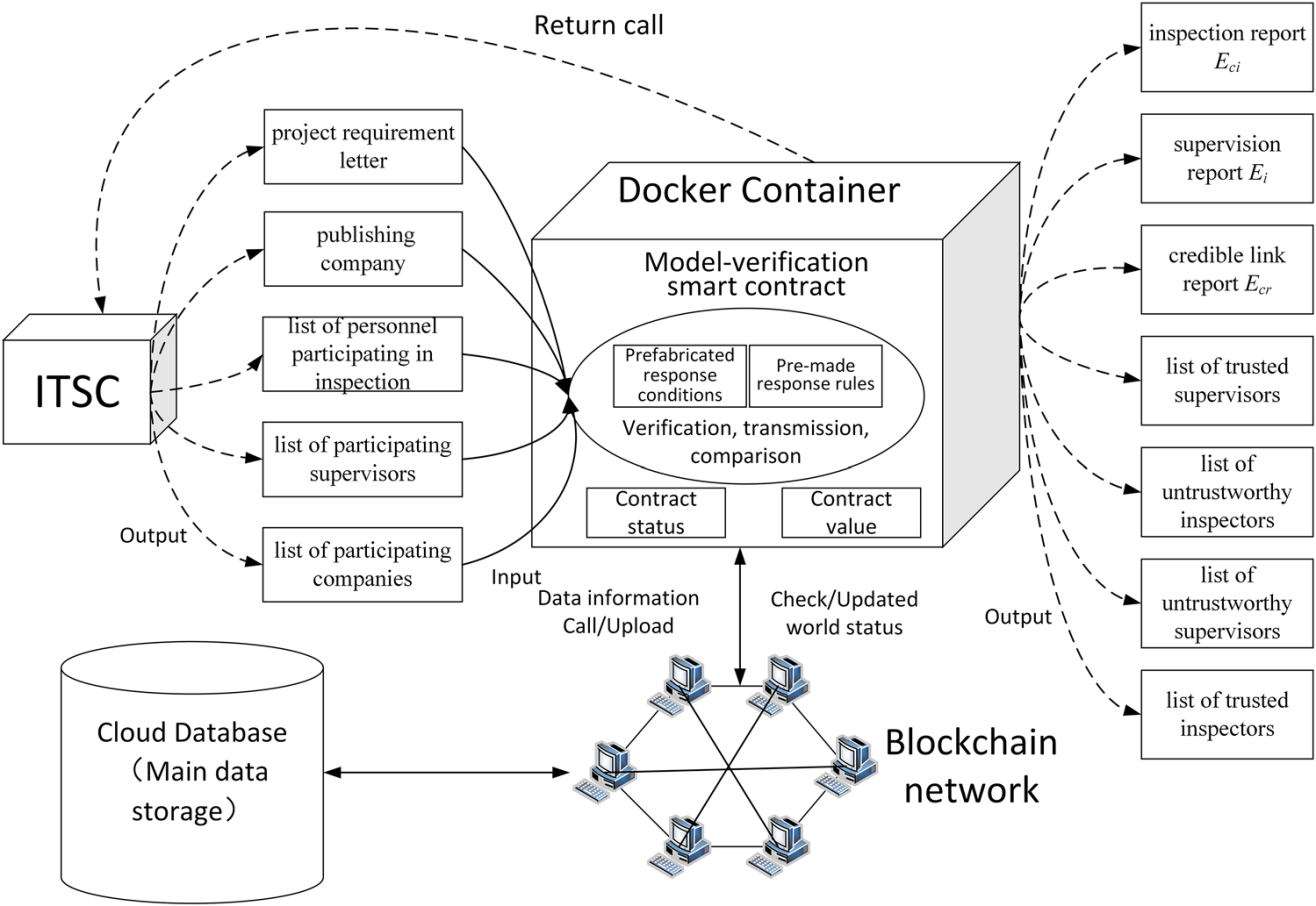
Logic diagram of the model-verification smart contract.

Finally, the contract quantifies the three dimensions of the data in the report, calculates and compares them through Euclidean distance, and screens out the lists of credible\untrustworthy supervisors and inspectors. The calculation of Euclidean distance is shown in Eqs. (13) and (14). The pseudo-code design of the model-verification smart contract is shown in Algorithm 2. The detailed logic design of Algorithm 2 is in Appendix A; Fig. 4 shows the contract logic diagram.

13 $$Dist(E_{ci} ,E_{i} ) = \sqrt {(W_{Ri} - W_{Ci} )^{2} + (Y_{Ri} - Y_{Ci} )^{2} + (Z_{Ri} - Z_{Ci} )^{2} } ,$$

14 $$Dist(E_{i} ,E_{cr} ) = \sqrt {(W_{Ri} - W_{cri} )^{2} + (Y_{Ri} - Y_{cri} )^{2} + (Z_{Ri} - Z_{cri} )^{2} } .$$

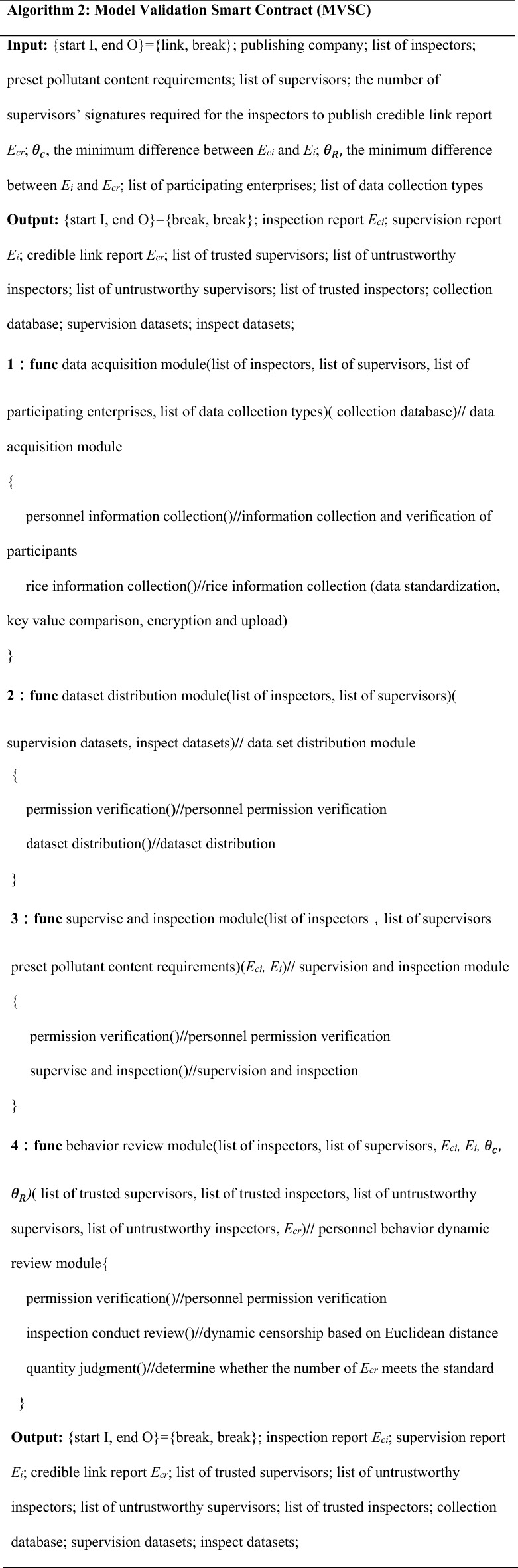

### Credit-evaluation smart contract

The contract in the model relies on the credit points of each participant and participating company to determine whether the person is eligible to participate in this batch of rice business. The evaluation criteria for credit points include the degree of completion of participating in the supervision/inspection process, whether there is fraudulent behavior, and the degree of trustworthiness of each process corresponding to the enterprise. The credit evaluation smart contract is custom designed to calculate and update the credit points between each participant. This contract can settle the contribution factor of the personnel participating in the inspection/supervision of this batch. And the contract guides the formation of the corresponding credible inspection database, credible supervision database and auxiliary inspection database. Based on the data exchange information between smart contracts and blockchain, the standard-link regional database is realized. The contract also guides the formation of a corresponding data model to provide a reliable source of data without verification for subsequent supervision.

The smart contract performs data classification and extraction based on the code information attached to the data and then performs model fusion based on the contribution coefficient of each participant. Equation (15) shows the fusion of trusted data supervision models. Equation (16) shows the fusion of the trusted inspection data model, Eq. (17) shows the fusion of the auxiliary inspection data model, and Eq. (18) shows the standard-link regional data model:

15 $$TR{ = }\sum {R_{suclist} } \sigma_{i} H_{i} ,$$

16 $$TC{ = }\sum {C_{suclist} } \sigma_{i} H_{i} ,$$

17 $$TP{ = }\sum {P_{suclist} } \sigma_{i} H_{i} ,$$

18 $$TE{ = }E_{cr1} { + }E_{cr2} { + }E_{cr3} { + }......{ + }E_{crn} = \left\{ {\begin{array}{*{20}c} {\sum\limits_{i = n} {W_{i} } } \\ {\sum\limits_{i = n} {Y_{i} } } \\ {\sum\limits_{i = n} {Z_{i} } .} \\ \end{array} } \right.$$

*TR* in Eq. (15) is a trusted supervision data model, and *R*_*suclist*_ is a list of trusted supervisors; $\sigma_{i}$ is the contribution coefficient for the supervisor, the value is the number of published supervisory reports *E*_i_. *H*_*i*_ is the unique identifier of the supervisor. In (16), *TC* is the credible inspection data model, and *C*_*suclist*_ is the list of credible inspectors and the contribution coefficient for the inspectors (except consumers); the value is the number of *E*_*cr*_ published credible link reports. *H*_*i*_ is the unique identifier of the inspector. In (17), *TP* is the auxiliary test data model, *P*_*suclist*_ is the list of trusted auxiliary testers (consumers), $\sigma_{i}$ is the auxiliary tester’s contribution coefficient; the value is the number of trusted link reports issued *E*_*cr*_. *H*_*i*_ is the unique identification of the inspector symbol. (The inspector refers specifically to consumers.) *TE* in Eq. (18) is the standard-link area data model, which is mainly a collection of data in all of the trusted link reports. Since the data are completely credible, the model can provide real conditions, such as pollution in all of the links of the rice supply chain.

**Figure 5:**
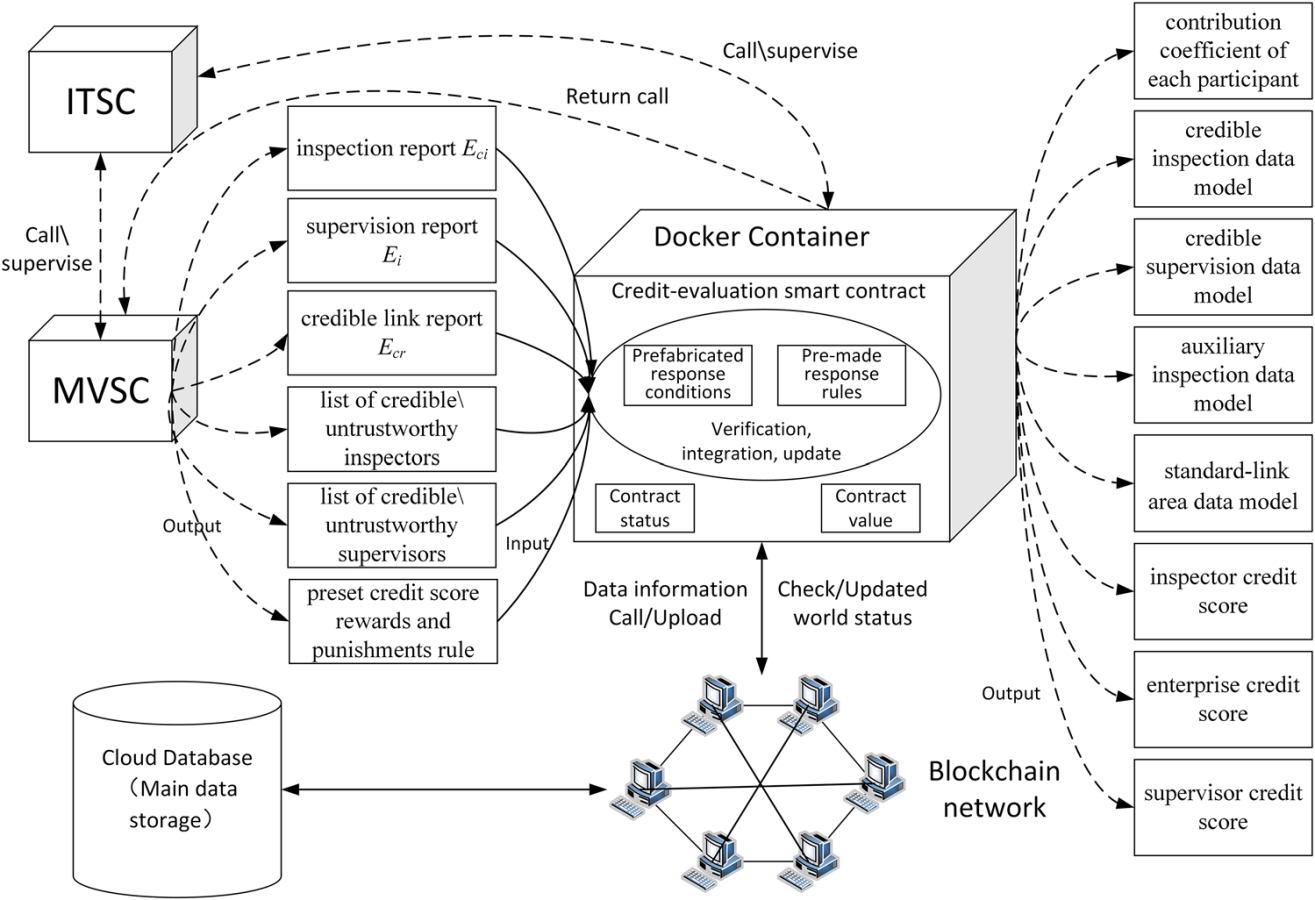
Logic diagram of the credit-evaluation smart contract.

The credit-evaluation smart contract updates its credit scores according to preset rewards and punishments based on the different lists of personnel participating in the business. The contract calculates the credit scores of related companies. The pseudo-code design is shown in Algorithm 3. The detailed logic design of Algorithm 3 is in Appendix A, and Fig. 5 shows the contract logic.
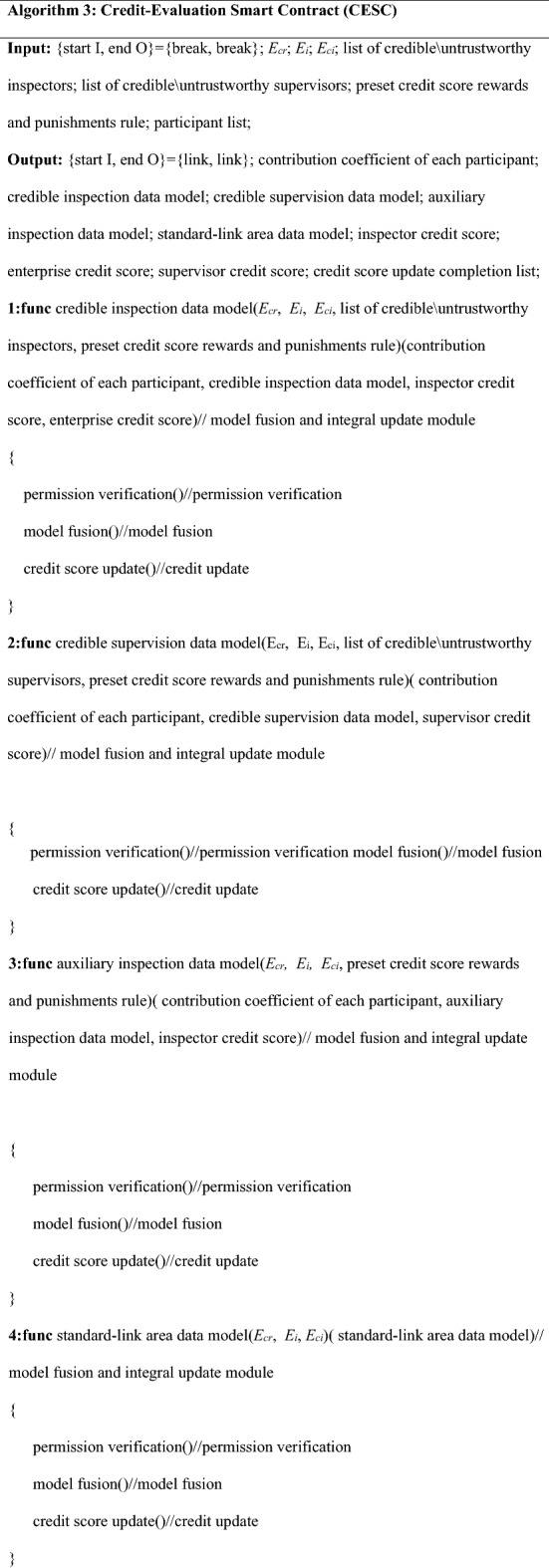

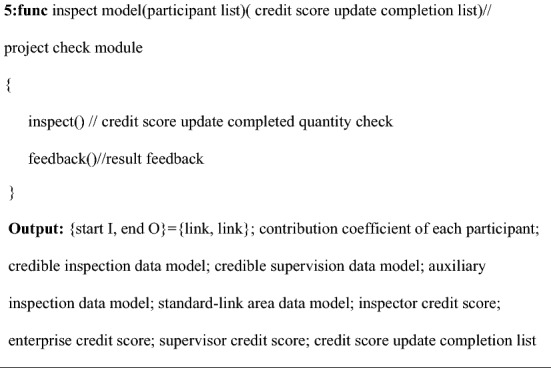

The iteration of a project in this model is achieved by initializing smart contracts, model-verification smart contracts, and mutual calling and supervision between smart contracts for credit evaluation. After the inspection and supervision of multiple projects, the dynamic supervision model of the rice supply chain is iterated repeatedly, the amount of reliable data continuously increases, and the supervision ability of the rice supply chain is improved.

## Results and analysis

### Analysis of model operation process

**Figure 6:**
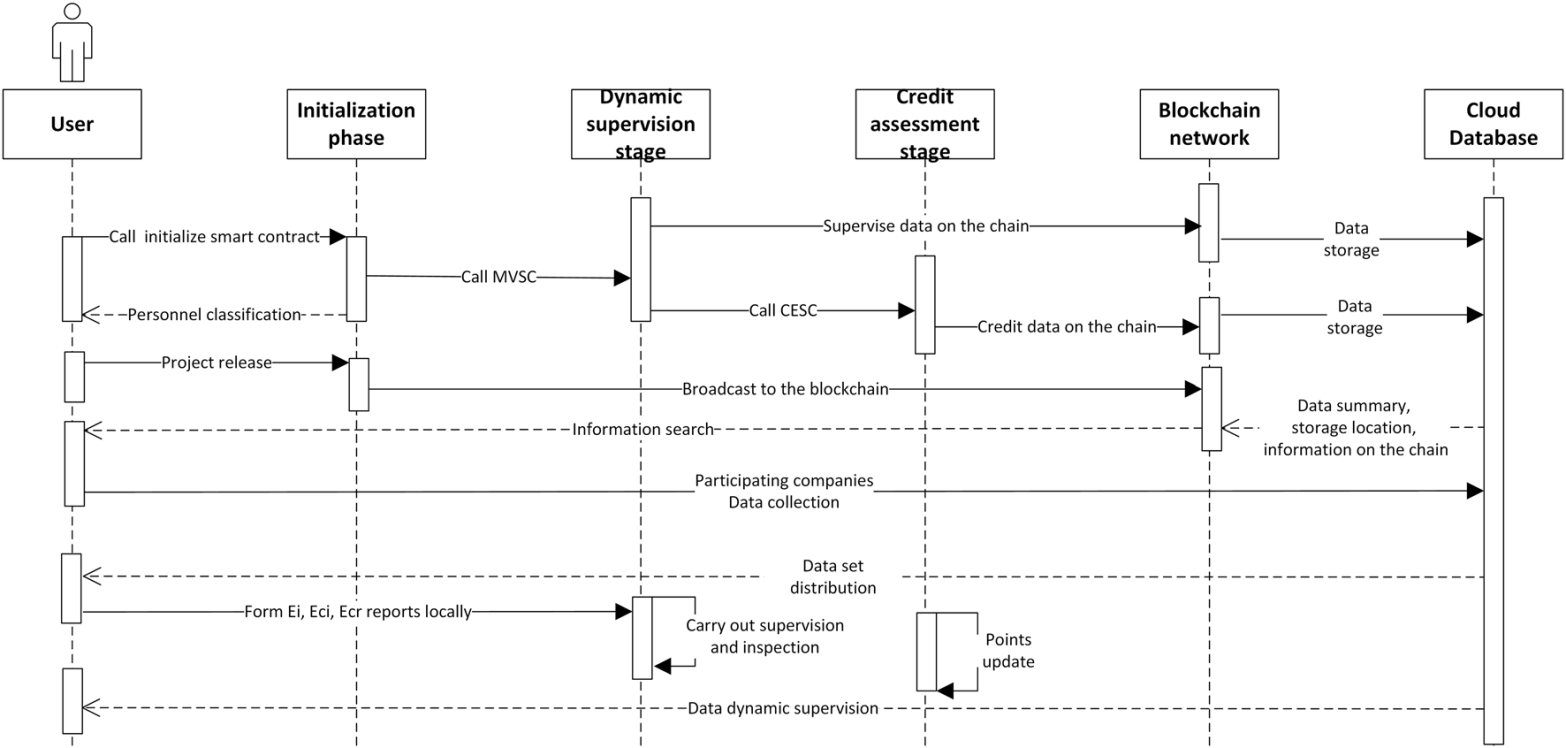
Operation sequence diagram of the dynamic supervision model for the rice supply chain.

The operation logic of the dynamic supervision model of the rice supply chain based on blockchain and smart contracts is mainly divided into three stages corresponding to the three smart contracts: the initialization stage corresponds to the initialization smart contract, the model-verification stage corresponds to the model-verification smart contract, and the credit evaluation stage corresponds to the credit-evaluation smart contract. A project runs through three stages into one iteration, and the model adopts a multithreaded method to solve the dynamic supervision of multibusiness rice. Figure 6 shows the running sequence diagram.

This model has a parallel mapping relationship with the blockchain network. The model first reaches node consensus, and each participant registers on the chain. The model is divided into three groups—participating companies, regulatory agencies, and consumers—based on the information collected by registration. After that, the model process is divided into three stages to achieve the dynamic real-time supervision of the whole life cycle of rice. The specific operation process is described below.

- Initialization stage

The sales company submits the project demand letter to the ITSC and applies for the release of the project. The project requirements include the required rice varieties, rice quality requirements, and the minimum credit score required to participate in the enterprise. ITSC verifies the credit points and business requirements of the sales company. After reaching the standards, the ITSC publishes the business on the blockchain through the application and assigns the company code *a*.

All kinds of enterprises inquire about the business instructions and apply to the ITSC to participate in the business. The ITSC checks the credit points of all of the enterprises. After the requirements are met, the ITSC passes the application and assigns each applying enterprise code 1, 2, 3.… Then, the list of participating companies is output. The regulatory authority applies to the ITSC to participate in the business, and the ITSC verifies the credit score of the regulatory authority. After reaching the standard, the contract assigns role code *b* and then outputs the list of supervision departments participating in this business. Except for the regulatory authorities, all personnel of participating companies can apply to the ITSC to become inspectors. The ITSC verifies applicants’ credit scores. After reaching the standard, it passes the verification, assigns role code *c*, and outputs the list of inspectors.

When participating companies, inspection personnel, and supervisors meet the requirements for multiple projects at the same time, each publishing company plays a game and competes for ownership of the participating personnel. The game uses the virtual regret minimization algorithm. The algorithm has the following advantages. First, it can reduce the computing resources required for the game to a large extent. Second, it enables players to adopt the optimal strategy for the game to infinitely approach the Nash equilibrium state and ensure the fairness of the game. Third, when the number of games is large enough, the winning times of each publisher will be infinitely close, which will keep unattended business from taking place.

(b) Dynamic supervision stage

After the initialization phase, the model enters the dynamic supervision phase. First, after initialization, the participants upload the corresponding link data to the blockchain network in real time. Inspectors and supervisors obtain relevant datasets, inspect and supervise data, and form corresponding inspection and supervisory reports. The supervisor checks the inspection report of the inspector and sends his or her own certification signature to the inspector after it is deemed qualified. When the inspector obtains sufficient certification signatures, he or she releases a report on the content of pollutants in the link and compares it with the supervision report to supervise the supervisor. If the supervisor shows dishonest behavior, his or her certification signature will be deleted and included in the list of dishonest supervisors. The credit points will then be deducted in the credit evaluation link.

The comparison of reports in the dynamic supervision phase uses the Euclidean distance algorithm to quantitatively analyze and calculate the three dimensions of data in the report. The model filters out the list of credible\untrustworthy inspectors, the list of credible\untrustworthy supervisors, and the standard-link area data list through comparative calculations. It then enters the credit evaluation stage.

(c) Credit evaluation stage

The operating logic of the credit evaluation stage is mainly encapsulated in the credit-evaluation smart contract. First, the model updates the credit scores of all of the participants based on the data in the dynamic supervision phase; that is, those on the trust list increase their points based on their contribution, and those on the untrustworthy list deduct their points. At this stage, the credit-evaluation smart contract performs data fusion on different data based on the fusion algorithm to form a trusted supervision data model, a trusted inspection data model, an auxiliary inspection data model, and a standard-link regional data model. Regarding providing trusted data sources for the dynamic supervision model, it can meet various real-world management needs, such as the internal management of regulatory agencies and internal management of enterprises.

### Prototype system verification

#### System architecture design

**Figure 7:**
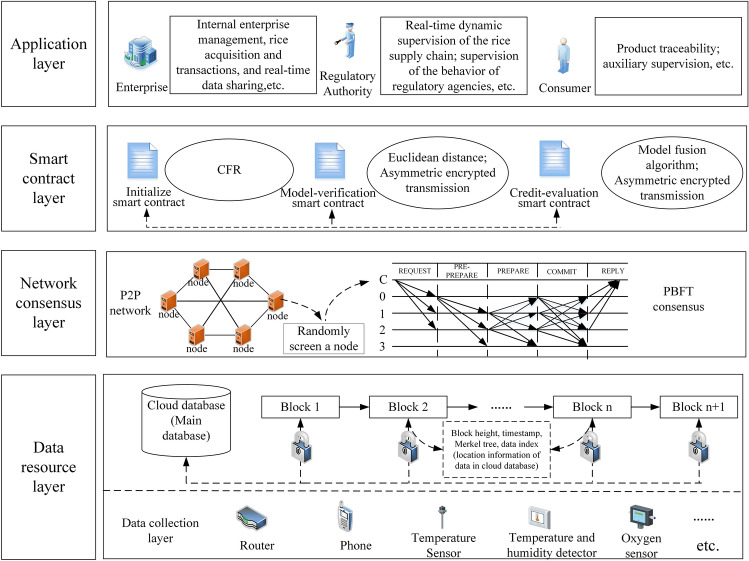
Prototype system architecture diagram.

This study constructed a prototype system based on the dynamic supervision model of the rice supply chain. Figure 7 shows the system architecture diagram. The system is divided into four layers: the application layer, smart contract layer, network consensus layer, and data resource layer.

The application layer serves various enterprises, regulatory agencies, and consumers throughout the supply chain. This layer provides functions such as internal enterprise management, rice acquisition and transactions, and real-time data sharing for enterprises in the chain. To jointly encapsulate the operating logic of the model, the smart contract layer is composed of the initialize smart contract, model-verification smart contract, and credit-evaluation smart contract. By designing related trigger conditions, the contract automatically executes the preset functions. The network consensus layer is a P2P network and a PBFT (Byzantine Fault-Tolerant Algorithm) consensus algorithm. Each node is connected in a point-to-point manner, and consensus is reached by randomly selecting a node, so that the nodes jointly elect a node as the master node. The data resource layer is divided into a storage layer and collection layer. The storage layer is composed of a blockchain network and a cloud database. The cloud database is the main data storage location, the index of data stored in the blockchain, and other information. The blockchain and the cloud data achieve the trusted interaction of data through an asymmetric encryption algorithm. The data collection layer is mainly for the real-time data upload of participating companies and supervisors who have been approved to participate in the business.

### Prototype system implementation

**Figure 8:**
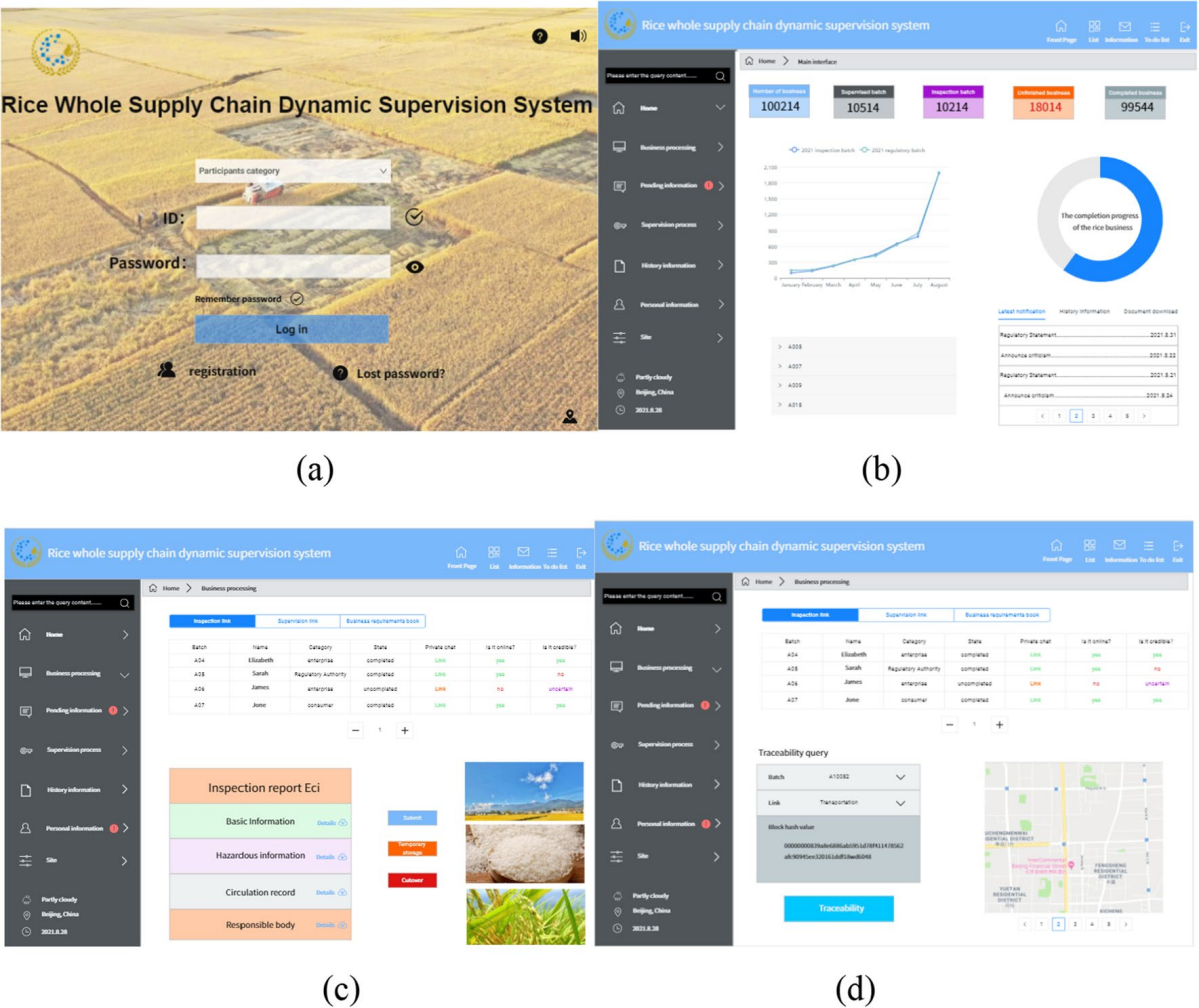
Prototype system interface diagramWe carried out simulation analysis on the prototype system of dynamic supervision of rice supply chain. The hardware environment is: the processor is Intel(R) Xeon Gold 6230 CPU@2.10 GHz 2.10 GHz (2 processors), the memory is 64.0 GB, and the hard disk is 8 T. Software environment: Linux version is 16.0.0, Ubuntu version is 20.04.1. Use Fabric 2.1 to build the prototype system, where the Docker version is 20.10.7, the docker-compose version is 1.25.0, and the Go language version is 1.17.2. Number of nodes: 10 supervisory nodes, 30 enterprise nodes, and 10 consumer nodes.

JAVA, Golang, and Python were used to develop the underlying blockchain of the system on the Hyperledger platform. QT was used for the front-end interface design, and RDS was used for off-chain data storage. Through the investigation of a batch of rice in North-east area, the data of this batch of rice was kept intact. With the participation of a supervisory unit, we used the batch of rice data to analyze and verify the practicability of the system. Figure 8 shows the dynamic supervision model for the rice supply chain. Figure 8a is the login interface; users can log in according to the different categories they belong to. Figure 8b is the main system interface. The left side is the menu bar, from which users can choose the corresponding functions. The top displays the system supervision, inspection, and processing business quantity. In the middle left is the current year’s inspection quantity and supervision quantity. The middle right displays the completion progress of the current supervision batch. The bottom left is the real-time game result of the publisher, who can view the supervision distribution information. The bottom right displays the latest notifications. Figure 8c is the local inspection interface for inspectors. The top part is the real-time data display table for the link, and the bottom part is the operation interface for link inspection and inspection report generation. Figure 8d is the traceability interface. The user selects the relevant batches and links. The system automatically matches the hash value of the relevant data and finally extracts the data from the cloud database for display. Verified by examples, the prototype system can supervise the data and personnel of the entire rice supply chain.

The proposed dynamic model for the rice supply chain is feasible. It can monitor pest information and the supervisory personnel of the rice supply chain in real time to ensure the timely detection of, timely resolution of, and precise accountability for rice quality and safety throughout the whole life cycle. The model provides a credible environment for its operation using blockchain technology. The model uses a decentralized architecture to enable data interconnection among various participants, breaking the traditional “island-style” mode of supervision. Table 4 shows a comparison of the traditional supervision model, a blockchain-based model, and a blockchain- and smart-contract-based model.

**Table 4:** Comparison of rice supervision methods.

Mode type	Coverage	Regulatory cost	Waste time	Regulatory credibility	Regulatory efficiency	Regulatory environment
Traditional supervision	Single link	High	High	Low	Low	Untrustworthy
Based on blockchain supervision	Full life cycle	Medium	Medium	Medium	Medium	Credible
Based on smart contract and blockchain supervision	Full life cycle	Low	Low	High	High	Credible

In traditional rice supply chain supervision, each link is separated. Therefore, there are information islands, so the traditional rice supervision is mainly reflected in the supervision of single-link data. For example, in rice consumption, Liu et al. used near-infrared spectroscopy to quickly identify fraudulent rice. To a certain extent, effectively alleviating the crime of rice fraud can serve to supervise the fraud of rice by the food regulatory agency^49^. However, the traditional supervision method is due to the centralized storage method. Therefore, its data maintenance cost is high, labor cost is high and time-consuming. And the centralized storage of pattern data is easy to lose, so its regulatory credibility is low. In addition, the degree of digitalization of traditional supervision methods is relatively low, its supervision efficiency is low, and the supervision environment is not credible. Compared with traditional supervision, blockchain-based supervision covers the entire life cycle. For example, the whole grain supply chain system architecture based on blockchain technology proposed by Zhang et al.^39^. And compared with the traditional supervision model, its supervision cost, time consumption, supervision reliability, supervision efficiency, etc. have been greatly improved. Most importantly, the characteristics of the blockchain itself make its supervision environment more credible. The supervision model based on blockchain smart contracts proposed in this paper uses smart contract technology to achieve automatic triggering and execution of the model, which improves the efficiency of model operation and supervision costs. The model also uses a virtual regret minimization algorithm, Euclidean distance, asymmetric encryption algorithm, and multisource data fusion algorithm to quickly process data and achieve the intelligent supervision of the entire supply chain. There are many advantages to smart contract-based regulation, but there are also some issues that need to be addressed. For example, its development difficulty is greater than that of traditional supervision models and blockchain-based supervision models.

### CFR algorithm analysis

**Figure 9:**
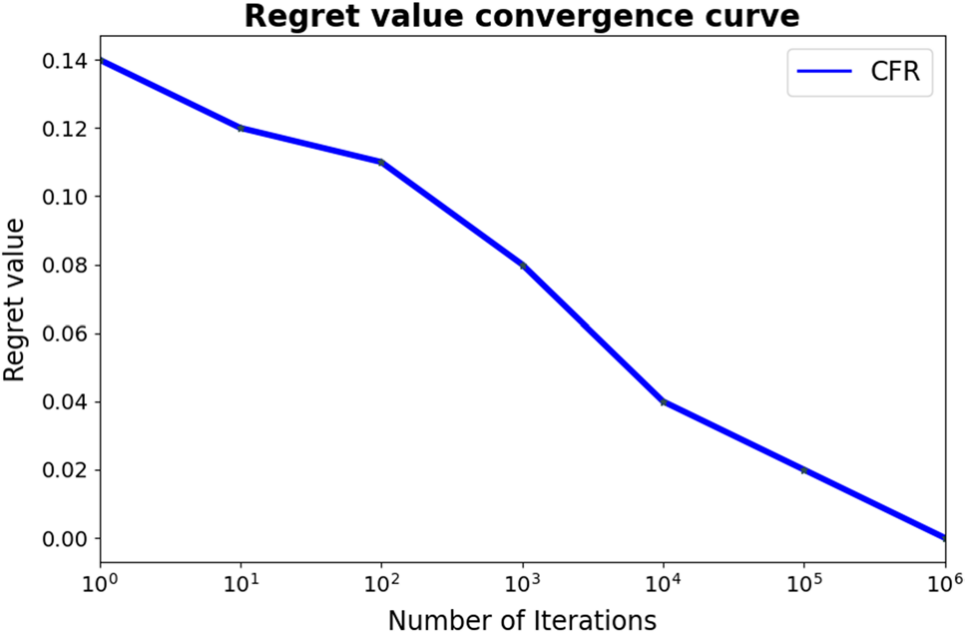
Algorithm performance analysis.

This study used the CFR algorithm to solve the concurrency problem of applying to participate in the business. Through the CFR algorithm, multiple business publishers can quickly reach or approach Nash equilibrium so that the game between business publishers can be fair to a certain extent. We analyzed the performance of the CFR algorithm. The algorithm uses two business publishers as an example to simulate and analyze the number of training iterations required to reach Nash equilibrium, as shown in Fig. 9. It can be seen that after 105 iterations of the algorithm, its regret value converges below 0.02. After 106 iterations, its regret value is infinitely close to 0; that is, it is infinitely close to Nash equilibrium.

**Figure 10:**
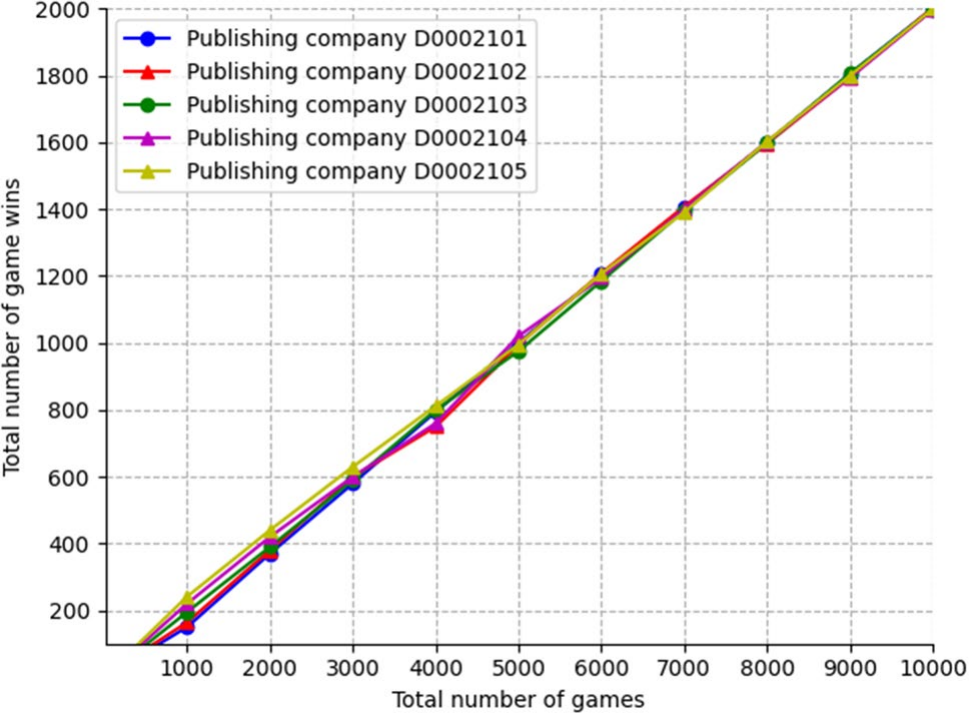
Game efficiency analysis.

We conducted a Python simulation for the rice supply chain. Figure 10 shows the situation of five publishing companies playing a game against the same applicant. It can be seen that as the number of games increases, the total number of winning games among the five publishing companies gradually tend to the same value. The average number of winning rounds is basically the same. The simulation verifies that the CFR algorithm can solve the concurrency problem of applicants in terms of practical application. However, its efficiency remains a problem. Improving the CFR algorithm so it can reach Nash equilibrium faster will be a key direction for optimizing this model.

### Supervision performance analysis

We tested the performance of the designed prototype system for the dynamic supervision of the rice supply chain. We randomly selected four batches of rice for testing, storage, transportation, and sales (Table 5). After the prototype system releases the rice demand of the enterprises and the initial testing of the participants and enterprises, each enterprise carries out real-time data inspection and supervision in the chain. Table 5 reveals that the system can complete the inspection and supervision of uploaded data within nine minutes and generate corresponding reports. The credit-evaluation smart contract can update the credit scores of participants in the chain in sufficient time.

**Table 5:** Dynamic supervision performance.

Batch	Link	Enterprise	Data collection completion time (day)	Supervision and inspection report generation time (min)	Credit score update completion time (ms)
A15240	Processing	Processing companies	12	6.2	2.25
A15261	Warehousing	Warehousing enterprise	3	7.5	3.35
A16284	Transportation	Logistics enterprises	1	8.6	2.21
A16250	Sales	Sales companies	36	4.5	1.25

### Analysis of model fusion efficiency

**Figure 11:**
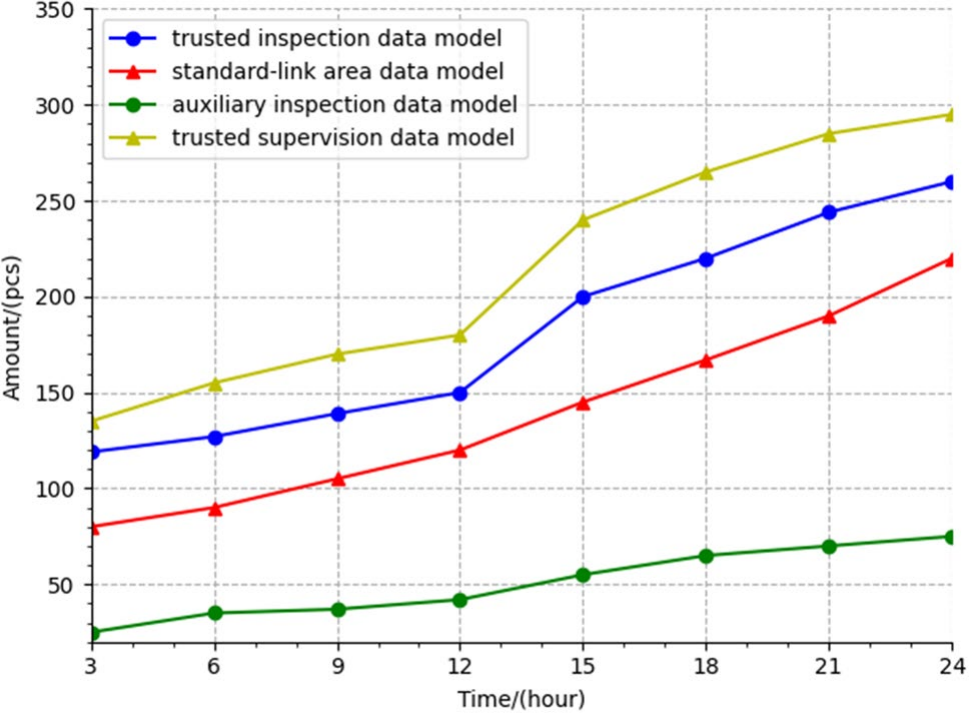
Analysis of trusted data sources.

**Figure 12:**
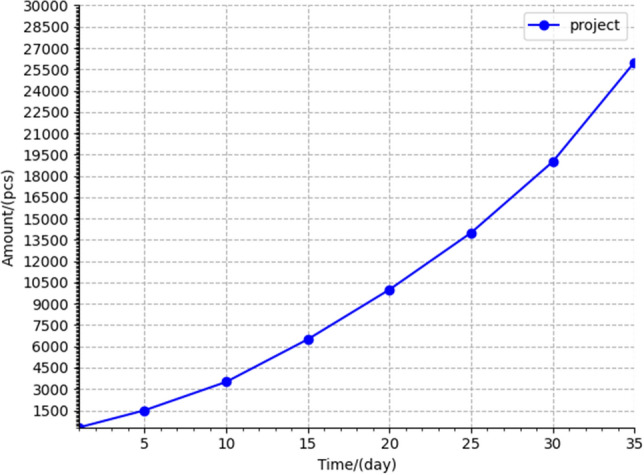
Business processing capability analysis of the prototype system.

The prototype system can be integrated to form four trusted databases. These store the trusted supervision data model, trusted inspection data model, auxiliary inspection data model, and standard-link area data model. We randomly collected data from the prototype system running for 1 day and analyzed it, as shown in Fig. 11. It can be seen that the number of models in the four databases steadily increases over time. With the increase of credible personnel in the four credible databases and repeated verifications of whether the behavior is credible, the dynamic supervision capability and efficiency of the prototype system for rice gradually increases (Fig. 12).

### Analysis of the role of trusted databases

**Figure 13:**
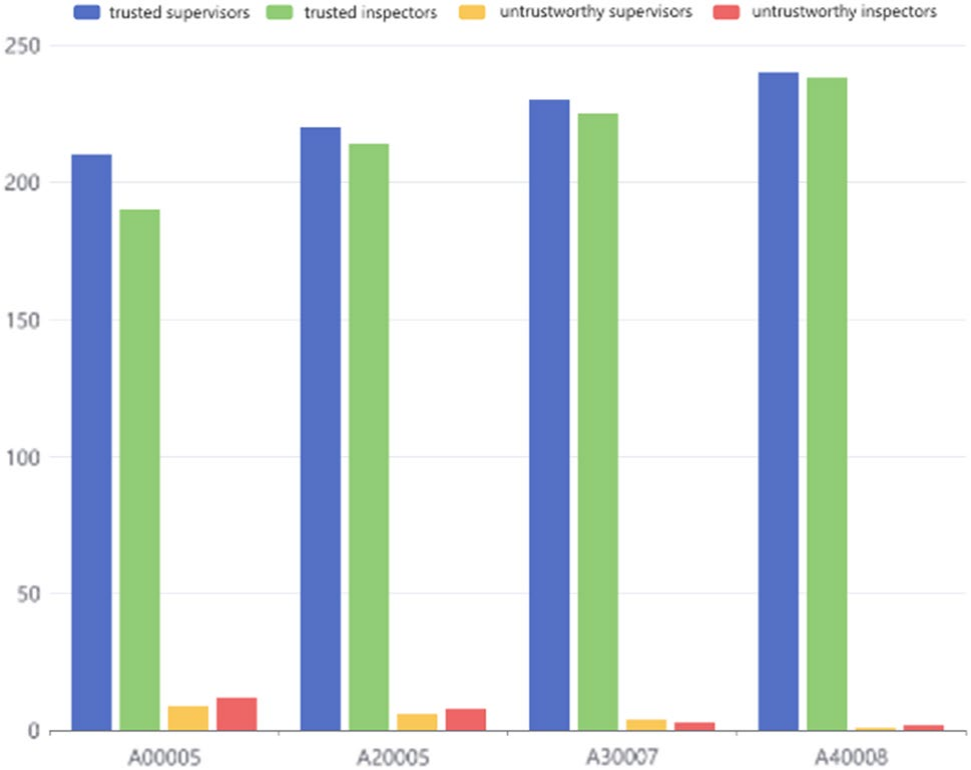
Analysis of dynamic supervision and inspection behavior.

With the continuous increase in trusted data models, the untrustworthy behavior of the prototype system in dynamic supervision and inspection continuously reduces with the increase in the number of processing services. In Fig. 13, we show the dynamic supervision and inspection behaviors of a certain link in the four batch stages of A00005, A20005, A30007, and A40008. It can be seen that with the continuous increase of rice business, the number of inspectors and supervisors with untrustworthy behavior continues to decrease. In the process of processing rice business, the trusted models in the database automatically formed through contracts gradually increase. When the system deals with rice business, its trusted personnel database is constantly increasing, and the credibility of the personnel involved in rice supervision business is continuously enhanced. Therefore, the untrustworthy behavior of the system in dynamic supervision and inspection is gradually reduced. The trusted data sources mined by this prototype system have a certain significance for optimizing rice supervision.

## Conclusion and future work

This study built a model based on blockchain and smart contracts to facilitate the dynamic supervision of the life cycle of rice supply chains. First, we analyzed the overall process of the rice supply chain and abstracted its main links. Then, we sorted out the participating roles in the entire supply chain, constructed a key data classification table for the supply chain based on its main links, and constructed an authorization data collection table based on each participating role. Next, we constructed a dynamic supervision model for the rice supply chain. Using smart contracts, we achieved the supervision of data in the supply chain and of supervisor behavior. We used the virtual regret minimization algorithm, asymmetric encryption algorithm, Euclidean distance, and multisource data fusion technology to achieve model functionality. Finally, we analyzed the correctness of the prototype system using an example. The results demonstrated the innovation of applying blockchain and smart contracts to rice supervision. The advantage of this approach is that it achieves interconnection and intercommunication among the data in all of the links while ensuring data security and achieving the dynamic supervision of the whole process. The proposed model can also provide credible data sources and solve the basic problem of untrustworthy blockchain data sources.

This study has certain limitations with regard to model operation speed and storage time, given the limitations of blockchain computing and storage resources. To overcome such problems, we will use cross-chain technology and a higher-configuration network in future work. Follow-up research can also seek to improve the CFR and encryption algorithms to reduce blockchain computing resources. The rice supply chain has different risk points than other grain and oilseed supply chains. It is mainly reflected in different links, different hazardous material information, different basic information, and different participating companies. The rice supply chain dynamic supervision model we constructed is suitable for the dynamic supervision of rice supply chain information. In the future, we will apply it to the dynamic supervision of other food crops to achieve the dynamic supervision of the entire food industry.

This research provides a feasible and practical solution for accelerating the digital transformation of the food industry, enhancing the ability to supervise food crops, and ensuring food security.

## Supplementary Information

Supplementary Information.

## Data Availability

All data generated or analysed during this study are included in this published article (and its Supplementary Information files).
