# Supplementary material for: Construction of rice supply chain supervision model driven by blockchain smart contract
Source: Sci Rep. 2022 Dec 5;12:20984. doi: 10.1038/s41598-022-25559-7 (PMC9722904; doi:10.1038/s41598-022-25559-7)
Supplement: Supplementary file 1 — Supplementary Information. [file 41598_2022_25559_MOESM1_ESM.docx]

Supplemental Material for:

^[[1]](#footnote-1)^Blockchain- and Smart-Contract-Based Supervision Model for the Rice Supply Chain

Xiangzhen Peng^a,b^, Xin Zhang^a,b,*^, Xiaoyi Wang^a,b,c^, Haisheng Li^a,b^, Jiping Xu^a,b^ and Zhiyao Zhao^a,b^

^a^ Beijing Key Laboratory of Big Data Technology for Food Safety, Beijing Technology and Business University, Beijing 100048, China

^b^ Key Laboratory of Industrial Internet and Big Data( Beijing Technology and Business University) ,China National Light Industry, Beijing 100048,China

^c^ Beijing Institute Of Fashion Technology, Beijing 100048, China

**S.1 Algorithm 1 Detailed Design**

| **Algorithm 1: Initialize Smart Contract (ITSC)** |
| --- |
| **Input:** {start I, end O}={link, link}; request; credit points of each participant (enterprises, supervisors, etc.)  **Output:** {start I, end O}={link, break}; project requirement letter; publishing company; list of personnel participating in inspection; list of participating supervisors; list of participating companies;  **1:** ITSC received the business release application from the sales company  Verify the credit points and project requirements of the issuing company  **If**  The credit score of the publisher is greater than the minimum credit score required to publish the project  The business requirement letter meets the requirements  **Return**  Give publishing company *a* unique code *a_i_* and broadcast the business requirements book to the blockchain network  **Else**  Dismiss the request  **2:** Other rice-related companies check the project requirements and apply to participate in the business  **If**  The company’s existing points are greater than the minimum points required for the company’s participation in the project  Verify that the number of companies is less than or equal to the maximum number of companies required for the project  **Return**  Application approved  **If**  The company has been verified by multiple publishing companies at the same time  **Return**  The publishing company conducts a fair game through the virtual regret minimization algorithm, and the winner obtains the right to participate in the company  According to the type of enterprise and the different links in which it is located, assign 1–5 codes  Output the list of companies successfully participating in the project  **3:** Participants apply to participate in the verification process  **If**  Participants are not supervisors and their credit points are greater than the minimum points required for the business  The number of people applying for participation is less than the maximum number required for the business  **Return**  Application approved  **If**  The participant has been verified by multiple publishing companies at the same time  **Return**  The publishing company conducts a fair game through the virtual regret minimization algorithm, and the winner obtains the right of inspection by the participant  Participants are given the identity of the inspection personnel and given code *b*  Export such personnel as a list of inspectors  **Else**  Dismiss the request  **4:** Supervisors apply for participation in supervision  **If**  Participating supervisors belong to the supervisor database  The credit score of the supervisor is greater than the minimum credit score required for the business  The number of supervisors involved in supervision is less than the number of supervisors required for the project  **Return**  Application approved  **If**  The supervisor has been verified by multiple publishing companies at the same time  **Return**  The publishing company conducts a fair game through the virtual regret minimization algorithm, and the winner obtains the right to participate in the company  Assign supervisor code c  Export the list of participating supervisors  **Else**  Dismiss the request  **5: Output:** {start I, end O}={link, break}, project requirement letter, publishing company, list of personnel participating in inspection, list of participating supervisors, list of participating companies |

**S.2 Algorithm 2 Detailed Design**

| **Algorithm 2: Model Validation Smart Contract (MVSC)** |
| --- |
| **Input:** {start I, end O}={link, break}; publishing company; list of inspectors; preset pollutant content requirements; list of supervisors; the number of supervisors’ signatures required for the inspectors to publish credible link report *E_cr_*;$\theta_{c}$, the minimum difference between *E_ci_* and *E_i_*; $\theta_{R},$ the minimum difference between *E_i_* and *E_cr_*;  **Output:** {start I, end O}={break, break}; inspection report *E_ci_*; supervision report *E_i_*; credible link report *E_cr_*; list of trusted supervisors; list of untrustworthy inspectors; list of untrustworthy supervisors; list of trusted inspectors; collection database; supervision datasets; inspect datasets;  **1:** Data collection  **If**  This person belongs to the participating companies, regulatory authorities, and consumers of this business  **Return**  Sort the data, upload it to the cloud database, and encrypt the index information and storage location on the chain  **2:** Get the dataset  **If**  The inspectors belong to the list of participating inspections  Supervisors belong to the list of participating supervision  **Return**  Encrypt the inspection dataset and distribute it to the inspector  Encrypt the supervision dataset and distribute it to the supervisor  **Else**  Dismiss the request  **3:** Carry out inspection and supervision  **If**  The inspectors belong to the list of persons participating in the inspection  Inspectors inspect the pollutant content of the link according to the preset pollutant content requirements  **Return**  The inspector submits the inspection report  Broadcast the report to other nodes  **If**  Supervisors belong to the list of participating supervisors  Supervisors monitor the pollutant content in the link according to the preset pollutant content requirements  **Return**  Supervisors submit supervision reports  **4:** Screen trustworthy\distrustworthy supervisors, trustworthy\distrustworthy inspectors  **If**  Supervisors belong to the list of trusted supervisors  **Return**  The inspection report is encrypted with the public key of the supervisor and distributed to the supervisor  **If**  Dist (E_ci_, E_i_) $\leq$θc  **Return**  The supervisor sends the certification signature to the inspector  **Else**  The supervisor rejects the pollutant content report submitted by the inspector  Go back to step 3  **5:** Screen trustworthy\distrustworthy supervisors, trustworthy\distrustworthy inspectors  **If**  The number of certified signatures ≥ the minimum number of signatures of supervisory personnel required by inspectors to inspect a single link  Go back to step 4  **Else**  The inspector is listed as an untrustworthy inspector  Go back to step 3  **6:** Screen trustworthy\distrustworthy supervisors, trustworthy\distrustworthy inspectors  **If**  Inspectors release credible link reports  **Return**  List the inspector as a trusted inspector  **7:**  **If**  dist (E_i_, E_cr_) ≥ θ_R_  **Return**  Back Add the supervisor to the list of untrustworthy supervisors  Remove the supervisor from the list of trusted supervisors  **If**  The number of credible inspectors and the list of untrustworthy persons is less than the number of persons participating in the inspection  **Return**  Go back to step 3  **8:**  **If**  The number of pollutant content reports in the credible link is greater than or equal to the minimum number required for the link  **Return**  {start I, end O}={break, break}  **Else**  Go back to step 3 of initializing the smart contract  **9: Output:** {start I, end O}={break, break}; inspection report *E_ci_*; supervision report *E_i_*; credible link report *E_cr_*; list of trusted supervisors; list of untrustworthy inspectors; list of untrustworthy supervisors; list of trusted inspectors; collection database; supervision datasets; inspect datasets; |

**S.3 Algorithm 3 Detailed Design**

| **Algorithm 3: Credit-Evaluation Smart Contract (CESC)** |
| --- |
| **Input:** {start I, end O}={break, break}; credible link report *E_cr_*; supervision report *E_i_*; inspection report *E_ci_*; list of credible\untrustworthy inspectors; list of credible\untrustworthy supervisors; preset credit score rewards and punishments rule; participant list;  **Output:** {start I, end O}={link, link}; contribution coefficient of each participant; credible inspection data model; credible supervision data model; auxiliary inspection data model; standard-link area data model; inspector credit score; enterprise credit score; supervisor credit score; credit score update completion list;  **1:** Reliable verification data model fusion and integral update  **If**  The inspector belongs to the list of trusted inspectors  The inspector is not a consumer  **Return**  Model fusion according to (15)  Increase its credit points and broadcast to other nodes  Add it to the list of updated credit scores  **If**  The inspector belongs to the list of untrustworthy inspectors  **Return**  Deduct their credit points and broadcast to other nodes  Add it to the list of updated credit scores  **2:** Reliable supervision data model fusion and points update  **If**  The supervisor belongs to the list of trusted supervisors  **Return**  Model fusion according to (16)  Increase its credit points and broadcast to other nodes  Add it to the list of updated credit scores  **If**  The supervisor belongs to the list of untrustworthy supervisors  **Return**  Deduct their credit points and broadcast to other nodes  Add it to the list of updated credit scores  **3:** Auxiliary data model fusion and integral update  **If**  The consumer belongs to the list of credible inspectors  **Return**  Model fusion according to (17)  Increase its credit points and broadcast to other nodes  Add it to the list of updated credit scores  **If**  The consumer belongs to the list of untrustworthy inspectors  **Return**  Deduct their credit points and broadcast to other nodes  Add it to the list of updated credit scores  **4:** Standard-link regional data model integration  **If**  Ecr reaches the preset number  **Return**  Model fusion according to (18)  **5:** Self-check  **If**  The number of people on the updated credit list is equal to the number of participants on the list  **Return**  Extract the inspectors belonging to a certain company based on the company code  Update corporate credit scores according to (1)  **Else**  Trace the credit points of participants  **6:** End of this project  **If**  The points are updated, and model fusion is completed  **Return**  Send part of the data to the publisher  End of this project  **7: Output:** {start I, end O}={link, link}; contribution coefficient of each participant; credible inspection data model; credible supervision data model; auxiliary inspection data model; standard-link area data model; inspector credit score; enterprise credit score; supervisor credit score; credit score update completion list |

1. Corresponding Author

   Email: zhangxin@btbu.edu.cn [↑](#footnote-ref-1)
